# *Salmonella* Typhi Porins OmpC and OmpF Are Potent Adjuvants for T-Dependent and T-Independent Antigens

**DOI:** 10.3389/fimmu.2017.00230

**Published:** 2017-03-09

**Authors:** Marisol Pérez-Toledo, Nuriban Valero-Pacheco, Rodolfo Pastelin-Palacios, Cristina Gil-Cruz, Christian Perez-Shibayama, Mario A. Moreno-Eutimio, Ingeborg Becker, Sonia Mayra Pérez-Tapia, Lourdes Arriaga-Pizano, Adam F. Cunningham, Armando Isibasi, Laura C. Bonifaz, Constantino López-Macías

**Affiliations:** ^1^Medical Research Unit on Immunochemistry, Specialties Hospital, National Medical Centre “Siglo XXI”, Mexican Social Security Institute, Mexico City, Mexico; ^2^Departamento de Inmunología, Escuela Nacional de Ciencias Biológicas, Instituto Politécnico Nacional, Mexico City, Mexico; ^3^Facultad de Química, Universidad Nacional Autónoma de México, Mexico City, Mexico; ^4^Institute of Immunobiology, Kantonsspital St. Gallen, St. Gallen, Switzerland; ^5^Immunity and Inflammation Research Unit, Hospital Juárez de México, Ministry of Health, Mexico City, Mexico; ^6^Facultad de Medicina, Departamento de Medicina Experimental, Universidad Nacional Autónoma de México, Mexico City, Mexico; ^7^Unit of R&D in Bioprocesses (UDIBI), Department of Immunology, National School of Biological Sciences, National Polytechnic Institute, Mexico City, Mexico; ^8^MRC Centre for Immune Regulation, College of Medical and Dental Sciences, Institute of Immunology and Immunotherapy, University of Birmingham, Birmingham, UK; ^9^Nuffield Department of Medicine, University of Oxford, Oxford, UK

**Keywords:** porins, adjuvants, influenza, Vi polysaccharide, IFN-gamma, IL-17, antibody responses

## Abstract

Several microbial components, such as bacterial DNA and flagellin, have been used as experimental vaccine adjuvants because of their inherent capacity to efficiently activate innate immune responses. Likewise, our previous work has shown that the major *Salmonella* Typhi (*S*. Typhi) outer membrane proteins OmpC and OmpF (porins) are highly immunogenic protective antigens that efficiently stimulate innate and adaptive immune responses in the absence of exogenous adjuvants. Moreover, *S*. Typhi porins induce the expression of costimulatory molecules on antigen-presenting cells through toll-like receptor canonical signaling pathways. However, the potential of major *S*. Typhi porins to be used as vaccine adjuvants remains unknown. Here, we evaluated the adjuvant properties of *S*. Typhi porins against a range of experimental and clinically relevant antigens. Co-immunization of *S*. Typhi porins with ovalbumin (OVA), an otherwise poorly immunogenic antigen, enhanced anti-OVA IgG titers, antibody class switching, and affinity maturation. This adjuvant effect was dependent on CD4^+^ T-cell cooperation and was associated with an increase in IFN-γ, IL-17A, and IL-2 production by OVA-specific CD4^+^ T cells. Furthermore, co-immunization of *S*. Typhi porins with an inactivated H1N1 2009 pandemic influenza virus experimental vaccine elicited higher hemagglutinating anti-influenza IgG titers, antibody class switching, and affinity maturation. Unexpectedly, co-administration of *S*. Typhi porins with purified, unconjugated Vi capsular polysaccharide vaccine (Vi CPS)—a T-independent antigen—induced higher IgG antibody titers and class switching. Together, our results suggest that *S*. Typhi porins OmpC and OmpF are versatile vaccine adjuvants, which could be used to enhance T-cell immune responses toward a Th1/Th17 profile, while improving antibody responses to otherwise poorly immunogenic T-dependent and T-independent antigens.

## Introduction

Numerous microbial components, such as CpG and flagellin, are pathogen-associated molecular patterns (PAMPs) that have been used as experimental vaccine adjuvants. Such activities reflect their capacity to activate innate immune responses through pattern recognition receptors, such as toll-like receptors (TLRs) (1). In addition to these PAMPs, the outer membrane proteins (OMPs, porins) of Gram-negative bacteria have also been shown to efficiently activate innate immune responses (2, 3). Porins are transport channels that play a key role in the diffusion of small molecules and in bacterial homeostasis (4). Numerous groups have studied the effects of porins from *Neisseria* (5), *Haemophilus* (6), *Pasteurella* (7), *Fusobacterium* (8), *Shigella* (9, 10), and *Salmonella* (11–14) on the activation of antigen-presenting cells (APCs). Because of the known effects that bacterial porins can have on the activation of APCs, some of these proteins have been used as potential vaccine adjuvants (5, 8, 13–15). The mechanism underlying the adjuvant effect of these porins includes events involving both innate and adaptive cells. For example, the adjuvant effect of PorB porin from *Neisseria meningitidis* is associated with the upregulation of costimulatory molecules and induction of lymphocyte proliferation, MHC-II overexpression, and secretion of pro-inflammatory cytokines by APCs, mediated mainly by TLR2/1 ligation (15–18).

*Salmonella* Typhi expresses multiple porins (19–21). While the major *S*. Typhi OmpC and OmpF porins are expressed constitutively, other porins, such as OmpS1 and OmpS2, are expressed at low levels under *in vitro* culture conditions and potentially during infection (22, 23). We have previously shown that the major and minor *S*. Typhi porins are highly immunogenic antigens and drive robust responses in the absence of exogenous adjuvants (14, 24–28). The major and minor *S*. Typhi porins can efficiently activate the innate immune system through canonical TLR2 and TLR4 signaling, resulting in increased costimulatory molecules and cytokine expression on dendritic cells (DCs) and B cells (12, 14).

Here, we evaluated the potential of the major *S*. Typhi porins to have an adjuvant effect against three distinct types of antigens, including ovalbumin (OVA) (a model antigen), an inactivated H1N1 2009 pandemic influenza virus, and a Vi polysaccharide, T-independent vaccine. These data show that porins promoted responses to all three antigens, indicating that these proteins may be used as adjuvants to a range of different types of antigens.

## Materials and Methods

### Ethics Statement

All animal procedures were conducted in accordance with national guidelines (Norma Oficial Mexicana, NOM-062-ZOO 1999), following review and approval by the Specialties Hospital Ethics Committee of Instituto Mexicano del Seguro Social (IMSS) (project number CNIC 2006-785-076).

### Antigens and Immunogens

Endotoxin-free OVA was purchased from Seikagaku Corp. (Tokyo, Japan). Pandemic influenza virus (strain A/Mexico/4482/2009(H1N1)) was grown in chicken embryos and kindly provided by the Instituto Nacional de Diagnóstico y Referencia Epidemiológicos (InDRE), Mexico City. The virus was inactivated using formalin [inactivated influenza A virus (iIAV)] and quantified by hemagglutination. For ELISA assays, iIAV was concentrated with 6% polyethylene glycol 8000 (Sigma, MO, USA) and further purified by centrifugation in a 20–60% sucrose density-gradient in NTE Buffer [100 mM NaCl, 10 mM Tris–Cl (pH = 7.4), 1 mM EDTA] as described elsewhere (29), and protein content was quantified by the bicinchoninic acid method.

Porins were purified from *S*. Typhi ATCC 9993 as previously described (12, 27). LPS content was measured using the limulus amebocyte lysate assay (Endosafe^®^ KTA; Charles River Endosafe Laboratories, Charleston, SC, USA), and all batches tested negative (detection limit, 0.01 ng LPS/10 μg protein). Digested porins (PorK) were prepared with 10 μg of proteinase K (New England Biolabs, MA, USA) per 30 μg of porins, followed by overnight incubation at 37°C and inactivation of the enzyme at 70°C for 1 h. LPS from *Escherichia coli* 0111:B4 was purchased from Sigma (MO, USA). Rehydragel was used as an alum control (Reheis, NJ, USA). Typhim Vi vaccine was obtained from Sanofi Pasteur (Lyon, France).

### Mice

Male BALB/c mice (6- to 8-week olds) were purchased from Harlan Laboratories (Mexico City, México). DO.11.10 mice OVA_323–339_ transgenic mice were bred at the animal facilities of the Experimental Medicine Department, Faculty of Medicine, Universidad Nacional Autónoma de México (UNAM).

### Immunization Protocol

BALB/c mice were immunized i.p. with 100 μg of OVA, 4 hemagglutinating units (HAU) of iIAV alone or with 10 μg of major *S*. Typhi porins, 10 μg of proteinase K digested porins, 5 μg of LPS, or 100 μg alum. Animals were boosted on day 15, and blood samples were taken at the indicated time points. For Vi CPS experiments, mice were immunized i.p. with 10 μg of Typhim Vi CPS vaccine alone or with 10 μg of *S*. Typhi porins, or proteinase K digested porins, with a boost on day 15.

### Adoptive Transfer and Assessment of T Cell Response

A total of 5 × 10^6^ CFSE-labeled CD4^+^ T cells from DO.11.10 mice were adoptively transferred i.v. After 24 h, mice were immunized s.c. in the footpads. Three days after immunization, the popliteal lymph nodes were extracted and CD4^+^ T cells were purified by negative selection with Dynabeads^®^ (Thermo Fisher Scientific, MA, USA). To assess *in vivo* T-cell proliferation, CD4^+^ T cells were stained with PE-conjugated KJ1-26 mAb (against DO.11.10 transgenic TCR) and APC-conjugated anti-CD4 (BD Biosciences, CA, USA). To determine the cytokine production, CD4^+^ T cells were cocultured at a ratio of 3:1 with splenic DCs purified by positive selection from naïve mice. DCs were used alone or pulsed with 100 μg of OVA. Then, 24 h after stimulation, the supernatants were collected, and the cytokines were quantified using a Th1/Th2/Th17 CBA kit following the manufacturer’s instructions (BD Biosciences, CA, USA). Data were acquired on a FACSCalibur (Becton-Dickinson, NJ, USA) and analyzed using FlowJo 7.5 software (Tree Star, Stanford, CA, USA).

### Antibody ELISA

High-binding, 96-well polystyrene flat bottom plates (Corning, NY, USA) were coated with 15 μg of OVA (Sigma, USA), 1 μg of iIAV or 1 μg of Typhim Vi vaccine per well, each dissolved in 0.1 M carbonate buffer (pH 9.5). Non-specific binding was blocked with 5% non-fat dry milk diluted in PBS pH 7.2. Sera were serially diluted twofold and incubated for 1 h at 37°C. Peroxidase-conjugated anti-mouse IgM, IgG H + L, IgG2a, IgG2b, or IgG3 (Invitrogen, CA, USA) were diluted at a ratio of 1:1,000. The plates were developed with 0.5 mg/mL ortho-phenylenediamine (Sigma, MO, USA) in 0.1 M citrate buffer (pH 5.6) containing 0.08% H_2_O_2_ (Sigma, MO, USA). The reaction was stopped with 1.25 M H_2_SO_4_, and the optical densities were read at 492 nm using an automatic ELISA plate reader (Multiskan Ascent, Thermo Scientific, Vantaa, Finland). The cutoff value was defined as threefold above the mean values of the negative controls.

High-avidity IgG antibodies were measured including a wash with a mild-denaturing agent to discriminate low-avidity antibodies, which are more likely to dissociate from the antigen–antibody complexes (30). Briefly, ELISA was performed as described above including a 10 min wash with 7 M urea solution after incubation of sera and before the addition of the secondary antibody.

### Hemagglutination Inhibition Assay

Sera were treated with receptor destroying enzyme (Denka Seiken, Tokyo, Japan) for 19 h at 37°C, according to the manufacturer’s instructions. Sera were serially diluted twofold in PBS using V-bottom plates (Nunc, Roskilde, Denmark). Diluted sera were incubated 30 min at RT with 8 HAU/25 μL of a pandemic influenza virus strain A/México/4482/2009 (H1N1). After incubation, 0.5% of chicken red blood cells were added to the plates and incubated 30 min at RT. The hemagglutination inhibition titer was established as the highest dilution of sera where hemagglutination was completely inhibited.

### Statistical Analysis

Statistical analysis was performed with GraphPad Prism 6.0 (GraphPad Software, La Jolla, CA, USA) applying one-way analysis of variance test with Bonferroni’s multiple comparison correction. *P*-values < 0.05 were considered significant. Significant differences are depicted as *P < 0.05, **P < 0.01, and ***P < 0.001.

## Results

### OmpC and OmpF from *S*. Typhi Are Effective Adjuvants for Promoting Antibody Responses to OVA

**Figure 1:**
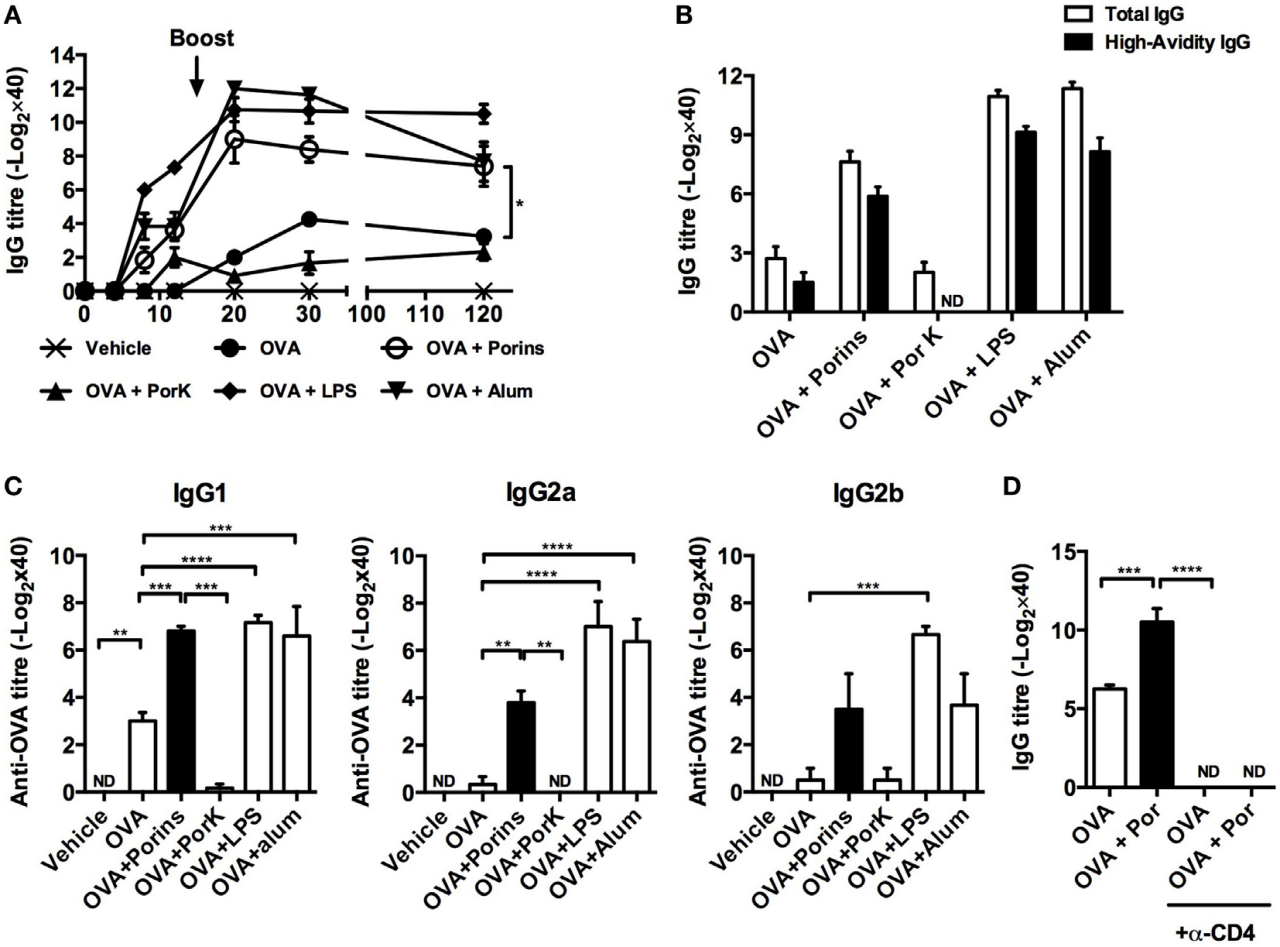
**OmpC and OmpF from *S*. Typhi promote antibody responses to ovalbumin (OVA)**. Four BALB/c mice per group were immunized i.p. with 100 μg of OVA, OVA + 10 μg of porins, OVA + 10 μg of PorK, OVA + 5 μg of LPS, or OVA + 100 μg of alum. **(A)** Total IgG antibody responses were measured at the indicated time points by ELISA **(B)** high-avidity IgG and **(C)** IgG1, IgG2a, and IgG2b were measured at day 30 post-immunization. **(D)** BALB/c mice were injected i.p. with a depleting anti-CD4 mAb (GK1.5) 3 days prior to immunization with OVA or OVA + porins. GK1.5 mAb was given every 3 days thereafter until day 20, and the serum IgG and IgM responses were measured by ELISA. Mean + SEM are shown. These data are representative of three independent experiments. Statistical analysis was performed using one-way ANOVA with Bonferroni test *post hoc*. For panel **(A)**, Student’s *t*-test was performed comparing only OVA versus OVA + porins groups. Statistical differences are depicted as *P < 0.05, **P < 0.01, ***P < 0.001.

To evaluate if major *S*. Typhi porins, hereafter referred to as porins, had an adjuvant effect on OVA antibody responses, we immunized BALB/c mice with OVA, OVA + porins, or OVA + proteinase K digested porins (PorK). Additionally, we included two groups immunized with OVA + alum and OVA + LPS to compare the relative effects of major *S*. Typhi porins to other known adjuvants. OVA-specific antibody titers were measured at different time points after immunization. OVA + porins induced higher IgG antibody titers, which remained detectable up to 120 days after immunization (Figure 1A), while OVA + PorK did not show an effect over OVA alone. The antibody titers induced by OVA + porins were similar to the levels induced by OVA + alum or OVA + LPS.

Next, we evaluated the affinity maturation and antibody class switching induced by co-immunization with *S*. Typhi porins at day 30 p.i. OVA + porins induced high-avidity antibodies compared to OVA alone (Figure 1B). Moreover, OVA + porins induced a significant response of IgG1, IgG2a, and IgG2b; the responses were abrogated when digested porins were used (Figure 1C). Antibody class switching and antibody titers induced by porins co-immunization were comparable to alum or LPS (Figure 1C).

Because CD4^+^ T cells cooperate with B cells to promote antibody responses (31), we investigated whether the adjuvant effect of *S*. Typhi porins was dependent on CD4^+^ T cells. Depletion of CD4^+^ T cells resulted in the complete abrogation of the antibody responses to OVA in the *S*. Typhi porins co-immunized group (Figure 1D), suggesting that CD4^+^ T cells are necessary for the adjuvant effect induced by *S*. Typhi porins on the antibody responses to OVA.

Altogether, these results suggest that *S*. Typhi porins can promote antibody responses to poorly immunogenic model antigens such as OVA, in a manner dependent on CD4^+^ T cell cooperation.

### *S*. Typhi Porins Can Increase OVA-Specific CD4^+^ T Cell Proliferation and Th1/Th17 Cytokine Production

**Figure 2:**
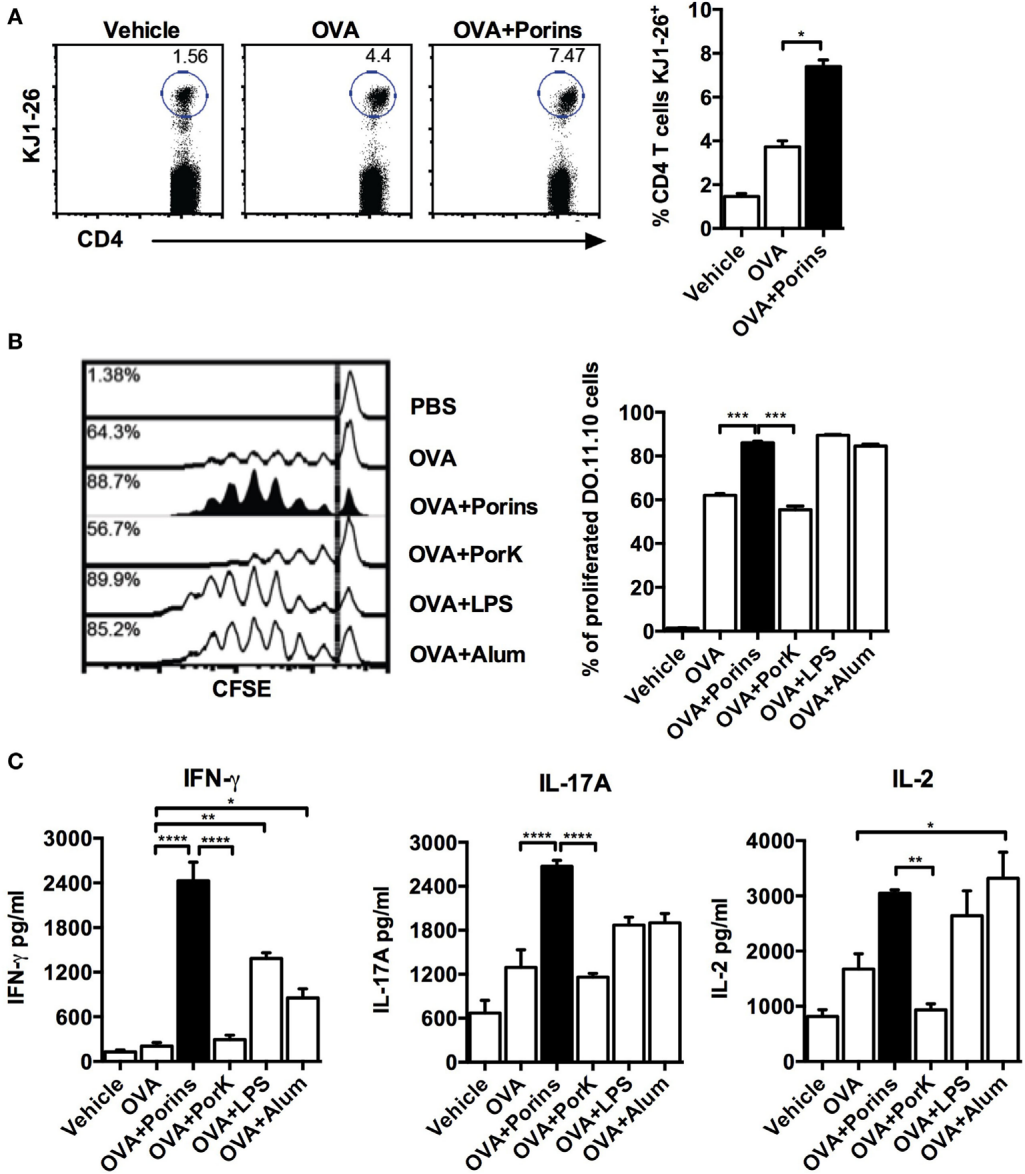
**Porins from *S*. Typhi increase cell proliferation and Th1/Th17 cytokine production by ovalbumin (OVA)-specific CD4^+^ T cells**. A total of 5 × 10^6^ CFSE-labeled CD4^+^ T cells from DO.11.10 mice were transferred to naïve BALB/c mice (4 mice per group) 24 h prior to s.c. injection into the front footpads with 100 μg of OVA, OVA + 10 μg of porins, OVA + 10 μg of PorK, OVA + 5 μg of LPS, or OVA + 100 μg of alum. Three days later, *in vivo* proliferation was assessed as frequency of OVA-specific cells (K1-J26^+^ cells) **(A)** and CFSE dilution **(B)**. **(C)** Coculture of purified CD4^+^ T cells from immunized DO.11.10 BALB/c chimeras with OVA-pulsed dendritic cells was performed, supernatants were collected 24 h later, and cytokines were measured by CBA assay. Mean + SEM are shown. These data are representative of three independent experiments. Statistical analysis was performed using one-way ANOVA with Bonferroni test *post hoc*. Statistical differences are depicted as *P < 0.05, **P < 0.01, ***P < 0.001.

Because we observed that the adjuvant effect of *S*. Typhi porins on antibody responses to OVA was dependent on CD4^+^ T cells, we examined if *S*. Typhi porins could also have an effect on OVA-specific CD4^+^ T cells. CD4^+^ T cells from DO.11.10 mice were CFSE labeled and adoptively transferred prior to immunization with OVA plus the different adjuvants. We found enhanced OVA-specific CD4^+^ T cell proliferation in the OVA + porins group, similar to alum or LPS (Figures 2A,B). In contrast, enhanced T cell proliferation was not observed with OVA + PorK. Assessment of cytokines produced by CD4^+^ T cells after *S*. Typhi porins co-immunization with OVA showed an increase in IFN-γ, IL-17A, and IL-2 (Figure 2C). Indeed, IFN-γ and IL-17A were highest in the OVA + porins group, while the OVA and OVA + PorK groups had similar cytokine profiles. These results show that *S*. Typhi porins enhance OVA-specific CD4^+^ T-cell proliferation as well as IFN-γ, IL-17A, and IL-2 production.

### *S*. Typhi Porins Promote Antibody Responses to Inactivated 2009 Pandemic Influenza Virus

**Figure 3:**
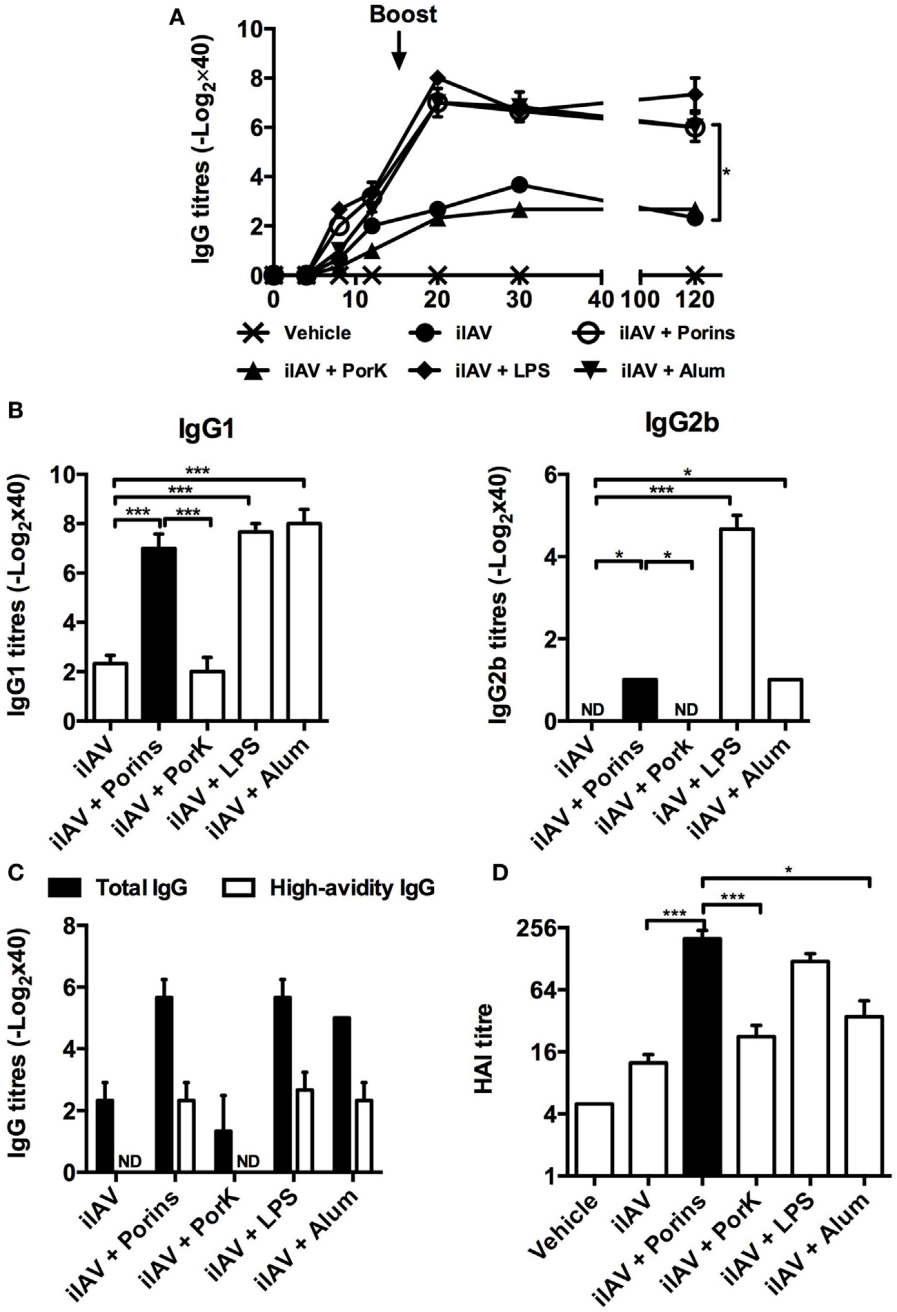
***S*. Typhi porins enhance functional antibody responses to an inactivated 2009 pandemic influenza vaccine**. Four BALB/c mice per group were immunized i.p. with 4 HAU of a pandemic inactivated influenza A virus (iIAV), iIAV + 10 μg of porins, iIAV + 10 μg of PorK, iIAV + 5 μg of LPS, or iIAV + 100 μg of alum. **(A)** Serum IgG antibody responses were measured at the indicated time points. **(B)** IgG1 and IgG2b, high-avidity IgG titers **(C)** and HI titers **(D)** were measured 30 days post-immunization by ELISA. Mean + SEM are plotted. These data are representative of three independent experiments. Statistical analysis was performed using one-way ANOVA with Bonferroni test *post hoc*. For panel **(A)**, Student’s *t*-test was performed comparing only ovalbumin (OVA) versus OVA + porins groups. Statistical differences are depicted as *P < 0.05, **P < 0.01, ***P < 0.001.

To evaluate if *S*. Typhi porins could improve the immune response to an experimental vaccine, we co-immunized mice with an inactivated pandemic influenza A (H1N1) virus (iIAV) plus *S*. Typhi porins, and we measured the antibody responses against influenza virus. We found that the addition of porins increased IgG antibody titers to iIAV, to a similar extent as alum or LPS, and that the response was maintained up to 120 days post-immunization (Figure 3A), with IgG1 being the most readily detectable isotype (Figure 3B). Additionally, we found an increase in high-avidity IgG titers (Figure 3C). A key element of any vaccine response is to enhance the protection afforded by immunization. Therefore, we examined the ability of the induced antibodies to promote agglutination of the virus. We found that the iIAV + porins group had higher titers of anti-hemagglutinin antibodies (Figure 3D) compared to iIAV and iIAV + alum and similar levels to the iIAV + LPS group. Together, these results show that *S*. Typhi porins can potentiate functional antibody responses to an experimental pathogen-derived vaccine.

### *S*. Typhi Porins Promote Antibody Responses to the T-Independent Anti-Typhoid Vi Antigen

**Figure 4:**
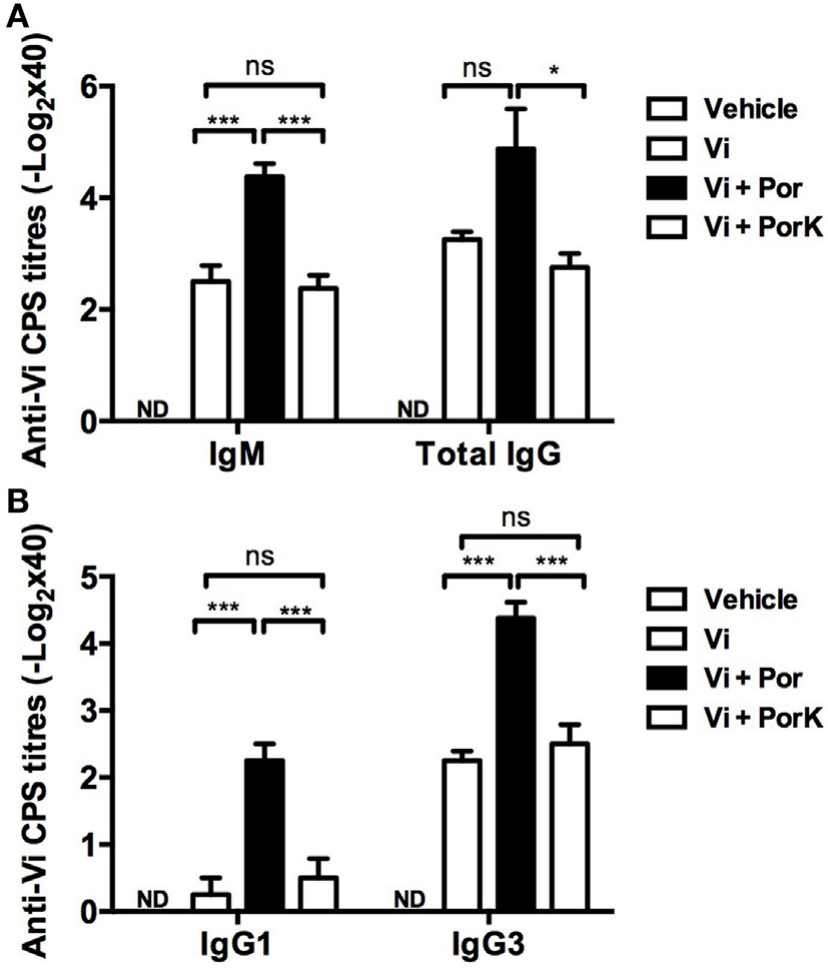
***S*. Typhi porins promote antibody responses to the T-independent anti-typhoid Vi antigen**. Four BALB/c mice per group were immunized i.p. with 10 μg of Vi CPS vaccine (Typhim), Vi + 10 μg porins or Vi + 10 μg of PorK on days 0 and 15. **(A)** IgM and total IgG responses and **(B)** IgG1 and IgG3 responses against Vi CPS were measured by ELISA on day 20 post-immunization. Mean + SEM are plotted. These data are representative of two independent experiments. Statistical analysis was performed using one-way ANOVA with Bonferroni test *post hoc*. Statistical differences are depicted as *P < 0.05, **P < 0.01, ***P < 0.001.

Because porins can induce T-dependent and T-independent responses (32), we examined if they could improve the immune response to a T-independent type 2 (TI-2) vaccine antigen. Mice were co-immunized with porins and the unconjugated CPS Typhim Vi vaccine, and the antibody responses against Vi CPS were evaluated. The addition of porins resulted in a fourfold increase in IgM and total IgG antibody titers compared to Vi CPS alone (Figure 4A). Surprisingly, co-immunization with *S*. Typhi porins only induced higher levels of both IgG1 and T-independent associated IgG3 isotype (Figure 4B). These effects of porins on the anti-Vi response were lost following the digestion of porins. Therefore, porins can improve the antibody responses to a TI-2 vaccine antigen.

## Discussion

Vaccination is one of the most efficient strategies to control infectious diseases. Whole inactivated microorganism-based vaccines are highly immunogenic; however, they frequently induce undesired side effects. To diminish this possibility, the development of vaccines has shifted toward the use of purified antigens. However, this strategy carries disadvantages, such as poor immunogenicity of the purified antigen (33). To overcome this problem, the use of adjuvants has been required to potentiate the immune response elicited by vaccination. Although increasing the magnitude of the immune response is essential for an adjuvant, the diversification and persistence of the response are other factors that should also be considered in the development of adjuvants to improve vaccine function (34).

Pattern recognition receptor activation of innate immune cells by adjuvants contributes to their function (35). Our previous studies have shown that *S*. Typhi porins can efficiently activate APCs and induce the expression of costimulatory molecules and cytokine production through TLR2, TLR4, and MyD88 signaling (12, 14). While minor *S*. Typhi porins possess adjuvant properties (14), the adjuvant potential of OmpC and OmpF porins has not been previously assessed.

*S*. Typhi porins enhanced the anti-OVA IgG antibody responses, which is otherwise a poorly immunogenic antigen, to a level comparable the antibody response seen when alum or LPS were added. Other porins have also shown an adjuvant effect on OVA antibody responses (8, 14, 36), although the persistence of this response has not been well examined in most of these studies. A persistent antibody response lasting several years has been shown in humans after immunization with porins (37). Further studies are needed to examine the nature of this persistent response.

Because long-lasting antibody responses and class switching toward IgG1, IgG2a, and IgG2b are associated with an involvement of T-cells (38) and because we observed that the adjuvant effect of *S*. Typhi porins was abrogated after treatment with an anti-CD4 monoclonal antibody, we analyzed how porins modified T-cell responses. We found that porins enhanced proliferation of OVA-specific CD4^+^ T cells. In addition, porins co-immunization promoted the production of IFN-γ, IL-17A, and IL-2 CD4^+^ T cells. This Th1/Th17-associated profile differs from the mixed Th1/Th2 response to the neisserial porin PorB, although this response was only examined in serum (15). Therefore, porins may be useful to modulate the immune response toward a Th1 or Th17 profile, and this property could be useful for vaccines that require Th1 responses to induce protection, such as *Mycobacterium tuberculosis* or *Chlamydia trachomatis* vaccines (39, 40). In addition, *S*. Typhi porins may enhance responses to vaccines where IL-17 responses are required. Other molecules can also have a Th1/Th17 promoting effect including CpG (41), poly I:C (42), and glucopyranosyl lipid adjuvant-stable emulsion (GLA-SE) (43) as well as *Borrelia burgdorferi* outer surface protein A (44) and *Brucella abortus* outer membrane protein 19 (45).

Porins had a remarkable effect on the response to the inactivated pandemic influenza virus H1N1 2009. Other TLR agonists with adjuvant properties, such as CpG (46), Pam3CSK4 (47), and GLA-SE (48), have been shown to improve the immunogenicity of inactivated influenza vaccines. Interestingly, the induction of HIA titers when major *S*. Typhi porins were included as an adjuvant was superior to the effect induced by alum, supporting the concept that it is not optimal to increase anti-influenza HIA titers (47).

Capsular polysaccharides (CPS), such as *S*. Typhi Vi, are TI-2 antigens and are B1b antigens in mice (49). Vaccination with such antigens elicits mainly IgM with limited class switching, affinity maturation, and immunological memory (50). Currently, most vaccines based on CPS perform well in healthy adults but have low immunogenicity in infants (51). The Vi CPS vaccine does not induce long-lasting immunity beyond 2 years (52). To overcome such a disadvantage, CPS are typically conjugated to a carrier protein to elicit T-cells and promote IgG switching and memory induction (52, 53).

*Salmonella* Typhi porins could enhance antibody responses to the purified, unconjugated Vi CPS, reflecting a similar finding when minor porins are used (14). Other antigens can also promote IgG responses, including PLc from *Vibrio cholerae* (54) and other bacterially derived antigens (55). Therefore, the presence of bacterial antigens may naturally augment such responses, possibly by adsorbing the Vi antigen. Porins can induce protective B1b antibody responses to *Salmonella* (32), and because Vi is also a B1b antigen (49), these effects could be a consequence of the stimulation of B1b cells by porins and Vi antigen. Thus, porins could be used as an adjuvant for TI-2 antigens, such as bacterial capsular polysaccharides.

The mechanism mediating the adjuvant effect might be related to the agonist effect of *S*. Typhi porins on TLR2 and TLR4 (Figure S1 in Supplementary Material). The activation of these receptors by porins might mediate the activation of APCs, such as DCs and macrophages. Activation of APCs then would induce the expression of costimulatory molecules and class II MHC expression and increase antigen presentation, as has been observed in DCs after stimulation or injection with porins (12, 14). It could also promote the secretion of pro-inflammatory cytokines, which in turn might activate additional cell populations, including T cells and B cells. Nevertheless, the effects of porins in B cells as a consequence of direct ligation of TLRs in these cells cannot be discarded, since in a previous report we showed that TLRs on B cells importantly contribute in shaping the antibody responses against porins (12).

Overall, our observations revealed that the adjuvant effect of major *S*. Typhi porins displayed two patterns. The first pattern indicated that the magnitude and diversity of the response were augmented similar to the model antigen OVA. The second pattern indicated that the diversity of the response elicited by the antigen alone remained unchanged but the magnitude of the response was augmented. This finding could mean that the mechanism underlying the adjuvant effect of porins might vary depending on the antigen that is co-immunized. This possibility should be explored further.

The adjuvant effect of porins is similar when compared to LPS; however, LPS cannot be used as adjuvant in animals and human vaccines due to its high toxicity. Contrary to LPS, we have previously shown that porins immunization is safe and well tolerated both for mice and humans (25, 27, 28); porins, therefore, represent an interesting alternative molecule that can mimic the adjuvant effect of LPS without carrying the same toxicity. Porins also induce, in terms of magnitude and duration of the response, a similar adjuvant effect than alum (the only worldwide used adjuvant in human vaccines). However, when comparing the features of the response induced by porins and alum, some differences can be spotted. For example, in DO.11.10 mice, immunization of porins is more efficient for driving IFN-γ and IL-17A-mediated T cell responses than alum. Likewise, porins induced higher hemagglutination-inhibiting antibody titers than alum when used as adjuvant for influenza virus immunization. Besides, we showed that porins potentiated the antibody responses against an unconjugated, Vi capsular polysaccharide vaccine, whereas alum has shown poor adjuvanticity when used to boost antibody responses against T-independent antigens (56). Taken together, we showed that porins induced a potent adjuvant effect similar to the induced by other well-known molecules with adjuvant properties such as LPS and alum. These results contribute to expand the knowledge about new choices of novel molecules that could be employed to improve vaccines or to develop new ones.

In conclusion, our results show that major *S*. Typhi porins OmpC and OmpF have adjuvant properties and potentiate, diversify, and extend B-cell responses to a diverse repertoire of antigens. They function in a manner associated with a Th1/Th17 profile. Our findings suggest that *S*. Typhi porins represent an additional choice of vaccine adjuvant for T-dependent and T-independent antigens.

## Author Contributions

MP-T performed the experiments, analyzed the results, and wrote the paper; NV-P and RP-P analyzed the results and wrote the paper; CG-C, CP-S, and MM-E designed the experiments and analyzed the results; IB and SP-T provided the reagents; LA-P, AC, and AI analyzed the results and revised the manuscript; and LB and CL-M designed the study, supervised the experiments, and revised the manuscript.

## Disclaimer

The results of this work have led to *Salmonella* Typhi OmpC and OmpF porins being patented to use them as adjuvants for vaccines.

## Conflict of Interest Statement

The authors declare that the research was conducted in the absence of any commercial or financial relationships that could be construed as a potential conflict of interest.

## Abbreviations

OMP, outer membrane protein; iIAV, inactivated influenza A virus; PorK, proteinase K digested porins; HAU, hemagglutinating units; GLA-SE, glucopyranosyl lipid adjuvant-stable emulsion.
