# Supplementary material for: Salmonella Typhi Porins OmpC and OmpF Are Potent Adjuvants for T-Dependent and T-Independent Antigens
Source: Front Immunol. 2017 Mar 9;8:230. doi: 10.3389/fimmu.2017.00230 (PMC5344031; doi:10.3389/fimmu.2017.00230)
Supplement: Supplementary file 1 [file Data_Sheet_1.DOCX]

**Supplementary material**

**Figure S1**. Porins are TLR2 and TLR4 agonists. HEK293 cells stably transfected with a plasmid coding for human TLR2, TLR4/MD2/CD14 or TLR5 were stimulated with porins (1µg/mL) for 24 h. IL-8 in supernatant was measured by ELISA. Means + SEM are plotted. Statistical analysis was performed using one-way ANOVA with Bonferroni test *post-hoc*. Statistical differences are depicted as **P<0.01, ns= non-significant.

**Supplementary figure methods**

HEK 293 cells stably transfected to express human TLR2/6, TLR4/MD2/CD14 or TLR5 were purchased from InvivoGen (CA, USA). Briefly, 2x10^5^ cells/well were stimulated with 1 µg/mL of porins, 1 µg/mL porins digested with proteinase K or 10 µg of LPS from *Escherichia coli* (Sigma, MO, USA), 10 µg of zymosan (InvivoGen, CA, USA) or 10 µg of flagellin from *Salmonella typhimurium* (InvivoGen, CA, USA) for 24 h. IL-8 production in supernatants was measured by ELISA using the OptEIA human IL-8 ELISA kit (BD Biosciences, CA, USA) following manufacturer’s instructions.
